# Use of quantitative PCR to assess the efficacy of albendazole against *Necator americanus* and *Ascaris* spp. in Manufahi District, Timor-Leste

**DOI:** 10.1186/s13071-018-2838-0

**Published:** 2018-06-28

**Authors:** Susana Vaz Nery, Jessica Qi, Stacey Llewellyn, Naomi E. Clarke, Rebecca Traub, Darren J. Gray, Andrew J. Vallely, Gail M. Williams, Ross M. Andrews, James S. McCarthy, Archie C. A. Clements

**Affiliations:** 1 Research School of Population Health, Australian National University, 62 Mills Rd, Canberra, 2601 ACT Australia; 2 Medical School, Australian National University, Building 42, Canberra, ACT 2601 Australia; 3 Clinical Tropical Medicine Laboratory, QIMR Berghofer Medical Research Institute, 300 Herston Rd, Brisbane, QLD 4006 Australia; 4 Faculty of Veterinary and Agricultural Sciences, The University of Melbourne, Parkville, Victoria 3052 Australia; 5 Kirby Institute, University of New South Wales, Wallace Wurth Building, Sydney, 2052 NSW Australia; 6 Present Address: Kirby Institute, University of New South Wales, Wallace Wurth Building, Sydney, 2052 NSW Australia; 7 School of Public Health, The University of Queensland, Corner of Herston Road and Wyndham Street, Brisbane, QLD 4006 Australia; 8 Menzies School of Health Research, Charles Darwin University, PO Box 41096, Casuarina, NT 0811 Australia

**Keywords:** Albendazole, Efficacy, *Necator americanus*, *Ascaris lumbricoides*, Hookworm, Soil-transmitted helminths, Anthelminthic drug efficacy

## Abstract

**Background:**

Soil-transmitted helminths (STHs) including *Ascaris lumbricoides*, *Necator americanus*, *Ancylostoma* spp. and *Trichuris trichiura* are cause of significant global morbidity. To mitigate their disease burden, at-risk groups in endemic regions receive periodic mass drug administration using anthelmintics, most commonly albendazole and mebendazole. Assessing the efficacy of anthelmintic drugs is important for confirming that these regimens are working effectively and that drug resistance has not emerged. In this study we aimed to characterise the therapeutic efficacy of albendazole against *Ascaris* spp. and *N. americanus* in Timor-Leste, using a quantitative polymerase chain reaction (qPCR) method for parasite detection and quantification.

**Results:**

A total of 314 participants from 8 communities in Timor-Leste provided stool samples before and 10–14 days after the administration of a single 400 mg dose of albendazole. Helminth infection status and infection intensity (measured in Ct-values and relative fluorescence units) were determined using qPCR. Efficacy was determined by examining the cure rates and infection intensity reduction rates. Albendazole was found to be highly efficacious against *Ascaris* spp., with a cure rate of 91.4% (95% CI: 85.9–95.2%) and infection intensity reduction rate of 95.6% (95% CI: 88.3–100%). The drug was less efficacious against *N. americanus* with a cure rate of 58.3% (95% CI: 51.4–64.9%) and infection intensity reduction rate of 88.9% (95% CI: 84.0–97.0%).

**Conclusions:**

The observed cure rates and infection intensity reduction rates obtained for *Ascaris* spp. and to a lower extent *N. americanus*, demonstrate the continued efficacy of albendazole against these species and its utility as a mass chemotherapy agent in Timor-Leste. Furthermore, this study demonstrates the usefulness of qPCR as a method to measure the efficacy of anthelminthic drugs. Additional research is necessary to translate Ct-values into eggs per gram in a systematic way.

**Trial registration:**

Australian and New Zealand Clinical Trials Registry 12614000680662 (registered 27 June 2014).

## Background

More than 1.4 billion people worldwide are estimated to suffer from infection with soil-transmitted helminths (STHs) [1]. The infective stages of these parasites thrive in the warm moist soils of tropical regions, and are transmitted through oral ingestion or skin penetration [2]. These modes of transmission mean that the most affected individuals are from poor communities that lack the adequate water, hygiene and sanitation necessary to prevent transmission and reinfection. *Ascaris lumbricoides* (roundworm), *Trichuris trichiura* (whipworm), and *Necator americanus* and *Ancylostoma* spp. (hookworms) are the most common STHs. They cause significant morbidity, particularly in children, who are commonly co-infected with multiple species [2]. Chronic infection can retard child growth and development, and infected individuals can suffer malnutrition, growth stunting and reduced cognitive abilities and intellectual capacity [2, 3]. Studies have indicated the significant adverse impact of STH infection on school attendance and performance and future economic productivity, although the health impact of STH infection is being debated [4–6].

The World Health Organization (WHO) has set goals to reduce STH-associated morbidity in children to a level at which it is no longer considered a public health problem [7]. To achieve this, populations at risk in endemic areas, mainly school-age children, are targeted with mass chemotherapy using anthelmintic drugs at either six monthly or yearly intervals depending on infection prevalence [8, 9]. The current recommended drugs are the benzimidazole drugs, albendazole and mebendazole [8], which are highly efficacious against *A. lumbricoides* with a recent meta-analysis indicating pooled cure rates of 95.7 and 96.2%, respectively, and egg reduction rates of 98.5 and 98.0%, respectively [10]. Albendazole is also efficacious against hookworm, with a pooled cure rate of 79.5% and egg reduction rate of 89.6%, compared to a cure rate of 32.5% and egg reduction rate of 61.0% for mebendazole [10]. Both drugs have poor efficacy against *T. trichiura*, with pooled cure rates of 30.7 and 42.1% for albendazole and mebendazole, respectively [10]. The WHO, international partners and pharmaceutical companies are committed to scaling up mass drug administration so that by 2020, 75% of at-risk children are being dewormed [7]. In 2016 alone, over 470 million schoolchildren were treated with anthelmintic drugs in endemic countries, corresponding to 69.5% of children at risk [11].

Greater usage of albendazole entails greater selection pressure of the drug for resistant parasite strains. Therefore, with the scaling up of mass drug administration programs, there are growing concerns over the potential for drug resistance to emerge in humans, similar to what has happened in other animals. In livestock, resistance to benzimidazoles is widespread, having emerged from the large-scale use of the drugs [12–14]. Monitoring the efficacy of anthelmintic drugs in order to detect the potential emergence of resistance in human populations is imperative to ensure that mitigation strategies can be promptly implemented to preserve the effectiveness of mass deworming campaigns [15]. STH are generally diagnosed using microscopy-based methods - most commonly the Kato-Katz method - to detect helminth eggs in stool. However, this method is known to have low sensitivity in lower-intensity and lower transmission settings, and requires examination of multiple samples to improve sensitivity [16]. Recently, quantitative polymerase chain reaction (qPCR)-based methods have been developed for the diagnosis and quantification of STH and validated as more sensitive than the conventional microscopy approaches [17–21].

The aim of this study was to determine the efficacy of a single dose of albendazole against STH infections using quantitative polymerase chain reaction (qPCR) for the detection and quantification of *Ascaris* spp., *N. americanus*, *Ancylostoma* spp. and *T. trichiura*, in the context of the implementation of the WASH for WORMS study, a cluster randomised controlled trial (RCT) in rural communities in Timor-Leste [17, 22]. Timor-Leste is a lower middle income country [23], where malnutrition and infectious diseases (such as pneumonia, diarrhea, malaria, tuberculosis and dengue) remain significant health problems [24]. The WASH for WORMS RCT which included community distribution of albendazole every 6 months for 2 years, at a time when no regular mass deworming was being implemented in the country [22]. The previous “*Lumbriga…Mak Lae Duni*” (Worms, no way!) mass drug administration program was implemented from 2005 to 2008 and was resumed in 2015. To our knowledge, this is the first albendazole efficacy study to be conducted in Timor-Leste and the first efficacy study to use qPCR for the calculation of cure rates and infection intensity reduction rates.

## Methods

### Study setting and data collection

This efficacy study was conducted from January 2012 to March 2013, in 8 communities in Manufahi municipality of Timor-Leste, which had been enrolled in the WASH for WORMS cluster RCT [22]. All community members were eligible for participation in the efficacy study, excluding women in the first trimester of pregnancy and children under 12 months of age. Baseline stool samples were collected for assessment of infection status and intensity, and individuals were subsequently given a single 400 mg dose of albendazole. Drug distribution was done by trained field workers. Children aged under 2 years were given half the dose. Between 10 and 14 days later, a second stool sample was collected to again determine infection status and intensity.

Sample size was calculated based on the following current recommendations [25]. Tree-based methods indicated that a minimum of 200 subjects (independent of infection status) is recommended to be able to detect a normal *vs* reduced efficacy based on faecal egg count reduction (FECR) [25]. Furthermore, WHO recommends a sample of 50 positive individuals for each parasite tested [26]. To achieve the necessary sample size and considering a compliance rate of 0.75 at each stool collection time point and estimated prevalences of 30% for *Ascaris* spp. and 50% for hookworm (based on studies in neighbouring Indonesia), we enrolled the first 8 of the 24 communities participating in the WASH for WORMS trial into the efficacy study, corresponding to approximately 500 eligible participants [27–29].

### Assessment of STH infection

Once collected, the stool samples were preserved at room temperature in 5% (weight/volume) potassium dichromate and transported to the QIMR Berghofer Medical Research Institute in Brisbane, Australia. The presence and intensity of protozoa and STH infection in stool samples was determined using qPCR methods as described previously [17]. In short, DNA extracted from samples that were spiked with a known amount of the plasmid used as positive control was run in a pentaplex real-time PCR reaction for detection and quantification of *Ascaris* spp., *N. americanus*, *Ancylostoma* spp. and *T. trichiura* [17]. The Rotor-Gene 6000 (Qiagen, Melbourne VIC, Australia) was used for all PCR assays [17]. Cycle threshold (Ct) values obtained using qPCR correspond to the amplification cycle at which the detected signal exceeds the background level. For a stool sample to be considered positive for infection, a limit of detection cut-off was set at 31 for *Ascaris* spp. and 35 for *N. americanus*, *Ancylostoma* spp. and *T. trichiura*, to ensure consistency with previously published PCRs [17]. For each qPCR assay, two runs were performed to generate two Ct-values. The arithmetic mean was taken of these two values to produce a single value. For calculation of intensity reduction rates, Ct-values were then converted to infection intensity measured in Relative Fluorescence Units (RFU) based on an assumed 100% reaction run efficiency, provided by the Rotorgene Q software (Infection intensity as determined by $\mathrm{qPCR}={10}^{-0.2980\kern0.05em \mathrm{Ct}+9.81}\ \mathrm{RFU}$) [17]. Samples which did not record a Ct-value were assigned an infection intensity value of 0.

### Statistical analysis

Pre- and post-treatment prevalence were compared using Chi-square test, or Fischer’s exact test in cases when frequency values were below 5. Only individuals who were positive at the pre-treatment time point were included in calculations of cure rate and infection intensity reduction rate derived from PCR.

Cure rate was calculated using the following formula:

$$ \frac{No. of\ individuals\ positive\  pre- treatment\ and\ negative\ post- treatment}{No. of\ individuals\ positive\  pre- treatment}\times 100 $$

95% binomial exact confidence intervals were calculated for both prevalence and cure rate. Age group (1–5 years; 6–11 years; 12–17 years; 18–64 years; and > 65 years) and sex were examined separately for potential associations with the probability of being cured, using the Wald Chi-square test adjusted for community-level clustering. The impact of baseline prevalence and baseline infection intensity (Ct-values) on cure rate was assessed using a multivariate logistic regression model, adjusted for age and sex, with a robust standard error adjusted for clustering at the community level.

Infection intensity reduction rate was calculated using the following formula as per WHO recommendations [25, 26]:

$$ \frac{\left( Arithmetic\ mean\ {intensity}_{pre- treatment}- Arithmetic\ mean\ {intensity}_{post- treatment}\right)}{Arithmetic\ mean\ {intensity}_{pre- treatment}}\times 100 $$

Confidence intervals for infection intensity reduction rate were calculated using a bootstrap re-sampling method with 10,000 replicates. The impact of baseline infection intensity (Ct-values) on infection intensity reduction rate was assessed using a multivariate linear regression model, adjusted for age and sex, with a robust standard error adjusted for clustering at the community level.

All analyses were conducted using Stata version 11.0 (College Station, TX, USA). A 5% significance limit was used for all analyses.

## Results

### Population under study

**Fig. 1:**
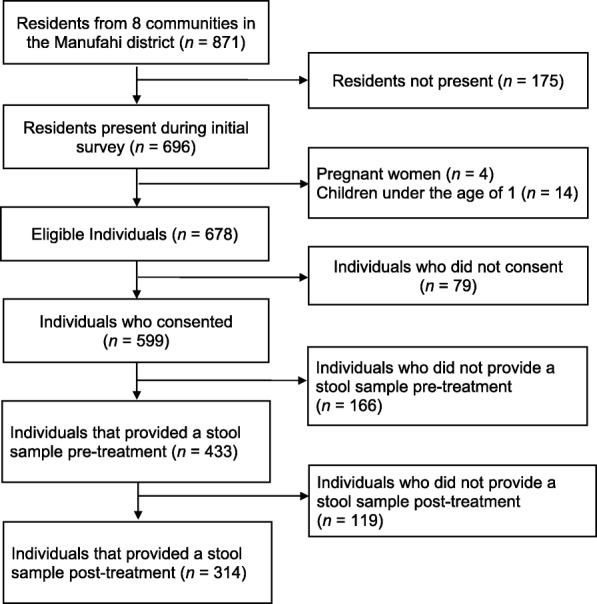
Efficacy study diagram

From the 8 communities enrolled, 678 individuals were present and eligible for study participation, of whom 599 (88.3%) agreed to participate. In total, 314 individuals provided both pre- and post-treatment stool samples and were included in the efficacy analysis presented here (Fig. 1). Participants ranged in age from 1 to 72 years, with a mean of 21 years. Of the study participants 54.8% were females and 45.2% were males.

The most prevalent species was *N. americanus* at 69.4% (95% CI: 64.1–74.2%), followed by *Ascaris* spp. at 51.6% (95% CI: 46.1–57.1%). *Ancylostoma* spp. (2.6%; 95% CI: 1.1–5.0%) and *T. trichuria* (1.3%; 95% CI: 0.3–3.2%) both had a low prevalence in the study population. For *Ascaris* spp., community-level prevalence ranged from 19.3% (95% CI: 10.9–31.8%) to 80.5% (95% CI: 65.3–90.0%), with a mean community-level prevalence of 54.2%. For *N. americanus*, community-level prevalence ranged from 52.5% (95% CI: 40.0–64.5%) to 87.8% (95% CI: 73.6–94.9%), with a mean community-level prevalence of 72.6%.

### Albendazole efficacy - cure rates and reduction in intensity

As shown in Table 1, the cure rate for *Ascaris* spp. was 91.4% (95% CI: 85.9–95.2%), and the cure rate for *N. americanus* was 58.3% (95% CI: 51.4–64.9%). The cure rate for *Ancylostoma* spp. was 100% (95% CI: 68.7–100%) and for *T. trichiura* was 50% (95% CI: 67.6–93.4%); due to the low number of individuals infected with *Ancylostoma* spp. and *T. trichiura,* further analyses were not performed for these helminths.

**Table 1:** Cure rates for *N. americanus* and *Ascaris* spp., overall and across sex and age groups

	*Necator americanus*				*Ascaris* spp.			
	*n*	Cure rate (%) (95% CI)	*χ* ^2^	*P*-value	*n*	Cure rate (%) (95% CI)	*χ* ^2^	*P*-value
Overall	218	58.3 (51.4–64.9)			162	91.4 (85.9–95.2)		
Sex			1.77	0.184			2.19	0.139
Male	104	54.8 (44.7–64.6)			74	89.2 (79.8–95.2)		
Female	114	61.4 (51.8–70.4)			88	93.2 (85.7–97.5)		
Age group (years)			1.88	0.758			5.71	0.222
1–5	35	60 (42.1–76.1)			40	92.5 (79.6–98.4)		
6–11	64	62.5 (49.5–74.3)			51	88.2 (76.1–95.6)		
12–17	29	58.6 (38.9–76.5)			22	95.5 (77.2–99.9)		
18–64	75	52 (40.2–63.9)			43	93.0 (80.9–98.5)		
65+	15	66.7 (38.4–88.2)			6	83.3 (35.9–99.6)		

*Abbreviations*: *n*, number of individuals positive pre-treatment; CI, confidence interval

Cure rates stratified by age group and sex are presented in Table 1. There was no significant difference in cure rate between males and females for either *N. americanus* (54.8% *vs* 61.4%, *χ*^2^ = 1.77, *df* = 1, *P* = 0.18) or *Ascaris* spp. (89.2% *vs* 93.2%, *χ*^2^ = 2.19, *df* = 1, *P* = 0.14). Similarly, age was not associated with being cured, with no statistically significant difference between age groups for either *N. americanus* (*χ*^2^ = 1.88, *df* = 4, *P* = 0.76) or *Ascaris* spp. (*χ*^2^ = 5.71, *df* = 4, *P* = 0.22).

For *N. americanus,* community-level baseline prevalence was negatively and significantly associated with cure: for a 1% increase in baseline prevalence, the odds of being cured decreased by 3% (OR = 0.97, 95% CI: 0.94–0.99, *P* = 0.03). Baseline infection intensity (Ct-value) was not associated with cure. For *Ascaris* spp., neither baseline community-level prevalence nor baseline infection intensity (Ct-value) were associated with cure. Age and sex were not associated with cure for either *N. americanus* or *Ascaris* spp. Full results of the multivariable analysis are shown in Table 2.

**Table 2:** Results of multivariate logistic regression for infection cure

	Odds ratio	95% CI	*P*-value
*Necator americanus*			
Baseline prevalence in community (%)	**0.97**	**0.94–0.99**	**0.029**
Baseline infection intensity (Ct-value)	1.04	0.94–1.14	0.474
Age (years)	0.99	0.98–1.01	0.463
Female sex	0.76	0.43–1.32	0.327
*Ascaris* spp.			
Baseline prevalence in community (%)	0.98	0.95–1.02	0.448
Baseline infection intensity (Ct-value)	1.02	0.94–1.12	0.596
Age (years)	1.00	0.97–1.03	0.975
Female sex	0.56	0.18–1.75	0.321

*Abbreviation*: CI, confidence interval

Bold indicates statistical significance

There was a significant decrease in infection intensity (RFU) for both STH species following treatment, with an infection intensity reduction rate for *Ascaris* spp. of 95.6% (95% CI: 88.3–100%) and 88.9% (95% CI: 83.0–97.0%) for *N. americanus* (see Table 3). The distribution of individual infection intensity reduction rates is shown in Table 4. In short, for both species the large majority of infections were cured or had an infection intensity reduction rate higher than 80%. An increase in infection intensity happened in 6.4% of the cases for *N. americanus* and in 1.2% of the cases for *Ascaris* spp.

**Table 3:** Infection intensity values before treatment, after treatment and reduction in infection intensity

	Pre-intervention mean infection intensity, RFU (95% CI)	Post-intervention mean infection intensity, RFU (95% CI)	Infection intensity reduction rate (%) (95% CI)
*N. americanus* (*n* = 218)	18,283 (13,251–23,316)	2024 (505–3,543)	88.9 (84.0–97.0)
*Ascaris* spp. (*n* = 162)	1,517,583 (1,124,742–1,910,424)	67,696 (0–200,926)	95.5 (88.3–100.0)

*Abbreviations*: *n*, number of individuals positive pre-treatment; RFU, relative fluorescent units

**Table 4:** Distribution of individual infection intensity reduction rates

Infection intensity reduction rate (%)	Number (%) of individuals	
	*N. americanus* (*n* = 218)	*Ascaris* spp. (*n* = 162)
100 (cured)	127 (58.3)	148 (91.4)
80–99.9	58 (26.6)	10 (6.2)
60–79.9	12 (5.5)	1 (0.6)
40–59.9	4 (1.8)	0
20–39.9	2 (0.9)	1 (0.6)
0–19.9	1 (0.5)	0
Increase in infection intensity	14 (6.4)	2 (1.2)

For *Ascaris* spp., baseline infection intensity (Ct-value) was not associated with infection intensity reduction rate. For *N. americanus*, a higher baseline infection intensity was associated with a higher intensity reduction rate (*P* = 0.04). There was no association between age or sex and infection intensity reduction rate for either species. Full results of the multivariate linear regression model are shown in Table 5.

**Table 5:** Results of multivariate linear regression for infection intensity reduction rate (%)

Variable	Regression coefficient	95% CI	*P*-value
*Necator americanus*			
Baseline infection intensity (Ct-value)	**-31.8**	**-61.5– -2.1**	**0.039**
Age (years)	-0.6	-2.2**–**0.9	0.371
Female sex	38.7	-6.6**–**84.1	0.083
*Ascaris* spp.			
Baseline infection intensity (Ct-value)	-0.20	-0.55**–**0.16	0.238
Age (years)	0.09	-0.30**–**0.48	0.594
Female sex	-9.90	-28.87**–**9.08	0.257

*Abbreviation*: CI, confidence interval

Bold indicates statistical significance

## Discussion

The findings of this efficacy study are consistent with earlier reports indicating that a single 400 mg dose of albendazole is highly efficacious against *Ascaris* spp. and less efficacious against hookworm [30]. While our cure rate for *Ascaris* spp. is comparable to previous reports, our detected cure rate for *N. americanus* was lower [30]. The lower cure rate obtained for *N. americanus* in this study is likely to be due to the higher diagnostic sensitivity of qPCR as compared to microscopy-based techniques that are generally used in efficacy studies, rather than implicating the existence of emerging benzimidazole resistance. That is, the lower sensitivity of microscopy relative to qPCR could mean that previously reported cure rates are over-estimated, as an individual may be misclassified as cured when in fact their faecal egg count was very low and not detected by microscopy. This is particularly true for hookworm, given that allowing the smear to stand for too long can result in the collapse and disappearance of hookworm eggs but not those of *Ascaris* spp. [31]

While cure rate is usually one of the indicators calculated in efficacy studies, it is not the best measure of drug efficacy, as it depends on baseline intensity and is influenced by the sensitivity of the diagnostic technique [32]. Therefore, current WHO guidelines recommend using a measure of intensity reduction - the faecal egg count reduction (FECR) - as the appropriate indicator of efficacy [26]. WHO guidelines stipulate that the FECR rate should exceed 95% in the case of *A. lumbricoides* and 90% in the case of hookworm [26, 33]. Because we employed qPCR as a diagnostic technique, infection intensity reduction rates were calculated based on PCR intensity values. Given that we were unable to also use microscopy methods in these samples we were not able to measure intensity in eggs per gram, hence the main limitation of this study is that our infection intensity reduction rate values are not directly comparable to FECR rates previously reported in the literature. However, given that both these parameters measure the proportional reduction in parasite load within a sample, and in the absence of a microscopic comparator allowing conversion of Ct to eggs per gram, we feel it is appropriate to apply thresholds pertaining to FECR rates to our results, as a first step in the use of qPCR for drug efficacy studies. Our infection intensity reduction rates were above reference efficacy thresholds for *Ascaris* spp. and just under the threshold for *N. americanus*, which suggest the continued efficacy of albendazole against these STH infections. In order for qPCR to be used as a quantitative method in efficacy studies where changes in infection intensity are the designated endpoints, additional research directly comparing infection intensity by microscopy and qPCR is needed. This will allow the conversion of Ct-values into eggs per gram, the establishment of appropriate thresholds for infection intensity reduction rates derived from qPCR to determine drug efficacy, and also the identification of qPCR intensity cut-offs corresponding to low, moderate and high intensity infection. In previous work infection intensity was derived by converting Ct-values in eggs per gram, using standard curves generated by qPCR assays undertaken on fresh hookworm and *Ascaris* spp. eggs. These were then used to interpolate eggs per gram from the PCR Ct-values obtained for the field samples [17]. Since this work was done, a number of potential confounding factors have been identified, including DNA extraction methods and stool preservative. A notable example is that we have observed that hookworm eggs preserved in potassium dichromate, as was the case in our field samples, can embryonate with storage, potentially resulting in an overestimation of infection intensity. Additional work is necessary to take into account storage conditions of field samples as was recently reported by Papaiakovou et al. [34]. Besides increased sensitivity, an additional advantage of the use of qPCR is that allows identification of the different hookworm species present in the population under study, which is not possible with microscopy. This will lead to a more refined understanding of albendazole efficacy against specific hookworm species, each of which can cause different levels and types of morbidity, and in the case of *A. ceylanicum* may require a One Health approach to overall control [35].

## Conclusion

As the first albendazole efficacy study to be conducted in Timor-Leste, the results of this study confirm the utility of this drug as a chemotherapeutic agent in the region. Furthermore, it demonstrates that qPCR can be effectively used to determine infection intensity reduction rates. In the future, this study will provide a useful point of comparison to establish whether there is any emerging resistance to albendazole in the context of mass chemotherapy campaigns that have recently restarted in Timor-Leste.
